# A case report and review of acute cholangitis with septic shock induced by *Edwardsiella tarda*

**DOI:** 10.1186/s12941-022-00524-4

**Published:** 2022-07-04

**Authors:** Yue Ding, Wanqi Men

**Affiliations:** Department of Clinical Laboratory, The Fourth Affiliated Hospital of Anhui Medical University, 372 Tunxi Road, Hefei, Anhui 230000 People’s Republic of China

**Keywords:** Acute cholangitis, *Edwardsiella tarda*, Bacteremia, Septic shock

## Abstract

**Background:**

*Edwardsiella tarda* (*E. tarda*) is a gram-negative facultative anaerobic bacterium. Gastroenteritis is the most common manifestation of *E. tarda* infection. However, parenteral infections can occur in immunodeficient hosts, as well as hepatobiliary diseases, malignancies, and/or diabetes. The prognosis of sepsis caused by *E. tarda* is very worse, with a mortality rate of 38%. We report the occurrence of acute cholecystitis with septic shock and *E. tarda* bloodstream infection.

**Case presentation:**

A 64-year-old male with acute cholecystitis secondary to hepatitis B virus infection showed fever and sudden upper abdominal pain. On arrival, right upper abdominal pain, nausea, vomiting, fever, and jaundice were observed. Computed tomography showed common bile duct stones and gallbladder stones. Choledocholithiasis with acute cholangitis was diagnosed and treated surgically. Due to septic shock, a blood culture was assessed showing *E. tarda* as the main pathogen. Choledocholithotomy, T-tube drainage, cholecystectomy, and intravenous antibiotic treatment after the operation. The patient recovered smoothly after the operation.

**Conclusions:**

Although *E. tarda* infection is extremely rare, it can cause rapid episodes of rapidly progressive and life-threatening disease, as well as intestinal and parenteral infections. If necessary, early surgical treatment of parenteral infection should be considered and antibiotics should be used in time.

## Background

*Edwardsiella tarda* (*E. tarda*) is a Gram-negative facultative anaerobic bacterium. Previous phylogenomic analysis revealed that *E. tarda* strains display two major highly divergent genomic types, EdwGI and EdwGII, and the former represents a genotype of fish-pathogenic isolates and is being recently proposed as a novel species of *E. piscicida* sp. nov. [1]. *E. tarda* is a rare human pathogen, that causes multiple infections that can cause gastroenteritis, wound infection, necrotizing fasciitis, and intrauterine infections [2]. *E. tarda* can also cause bloodstream infections and septic shock, with a poor prognosis. The mortality of septicemia caused by *E. tarda* infection is 38% [3–6]. Therefore, *E. tarda* bloodstream infections are dangerous and acute, which is relevant in immunocompromised patients. If they are not treated in time, the patients may become life-threatening [7]. Due to the fewer reported cases of bloodstream infection of *E. tarda*, here we report the occurrence of acute cholecystitis septic shock associated with *E. tarda* blood infection in a 64-year-old man with choledocholithiasis secondary to hepatitis B virus infection.

## Case presentation

The patient, a 64-year-old man, was admitted to the hospital’s general surgery department with right upper abdominal pain for 3 days with chills and fever. The patient was suffering from pain in the right upper abdomen with nausea and vomiting, accompanied by chills and fever, yellow eyes and urine, a maximum body temperature of 39 °C, moderate yellowing of the skin, and mucous membranes all over the body. The breath sounds of both lungs were thick, and no obvious dry and wet rales. Diagnosed as common bile duct stones with acute cholecystitis, septic shock, obstructive jaundice, hepatitis, and bronchiectasis with infection. Under general anesthesia in the emergency department, the operation was successful with choledochotomy stone drainage, T-tube drainage, and cholecystectomy. Due to the patient’s older age, poor general condition, hemodynamically unstable, and septic shock before surgery, he was transferred to the intensive care unit that night to strengthen monitoring and treatment. Blood test results on admission (Table 1).

**Table 1:** Results of blood tests

	Admission	Discharge	Reference
WBC	11.89 × 10^9^/L	5.16 × 10^9^/L	4.00–10.00 × 10^9^/L
RBC	2.81 × 10^12^/L	4.11 × 10^12^/L	4-5.5 × 10^12^/L
HB	90 g/L	122 g/L	120–160 g/L
PLT	54 × 10^9^/L	104 × 10^9^/L	100–300 × 10^9^/L
Na	138.7 mmol/L	140.3 mmol/L	137–145 mmol/L
K	4.35 mmol/L	4.41 mmol/L	3.5–5.1 mmol/L
CL	107.4 mmol/L	105.2 mmol/L	98–107 mmol/L
Ca	2.07 mmol/L	2.32 mmol/L	2.1–2.55 mmol/L
CRP	91.4 mg/L	4.0 mg/L	0–10 mg/L
TP	50.4 g/L	60.5 g/L	63–82 g/L
ALB	29.4 g/L	32.7 g/L	35–50 g/L
TBIL	87.2µmol/L	25.3µmol/L	3–22µmol/L
ALT	592 U/L	85 U/L	0–40 U/L
AST	945 U/L	94 U/L	0–30 U/L
PT	17.8 S	12.4 S	9-14 S
APTT	51.9 S	33.8 S	21.1-36.5 S
PCT	31.83	1.52	0-0.1ng/ml

*WBC* white blood cells, *RBC* red blood cells, *HB* hemoglobin, *PLT* platelets, *Na* sodium, *K* potassium, *CL* chloride, *Ca* calcium, *CRP* C-reactive protein, *TP* total protein, *ALB* albumin, *Glu* glucose, *TBIL* total-bilirubin, *AST* aspartate aminotransferase, *ALT* alanine aminotransferase, *PT* prothrombin time, *APTT* activated partial prothrombin time, *PCT* procalcitonin

To ameliorate the anemia and improve the blood coagulation function, the patient was infused with 2U de-suspended red blood cells and 200 mL of frozen plasma. The patient’s symptoms improved after the blood transfusion.

The next day, the patient’s body temperature is 37.4 °C, a little white sputum. A little bloody liquid was drained from the drainage bag connected to the right abdominal drainage tube, a little dark green bile was drained from the drainage bag connected to the T tube, and the common bile bacterial culture was added for drug sensitivity detection. Routine blood results showed an increase in white blood cells from 11.89 (10^9^/L) to 22.17 (10^9^/L), a rise in ultrasensitive C-reactive protein (CRP) from 91.4 mg/L to 159.32 mg/L, and an elevation in the percentage of neutrophils from 93.3 to 94.2%. Because the patient’s infectious index was very high and progressively increased with the number of days of hospitalization. The biliary tract infection was very heavy, complicated by septic shock and liver function injury. Imipenem cilastatin (1 g intravenous drop Q8h) was given to strengthen anti-infection treatment.

The patient’s body temperature did not decrease on the 3rd day. He coughed up more white mucus. More bloody fluid and brown bile flowed out of the drainage tube and continued to be sent for common bacterial culture of bile and bilateral double-bottle blood culture of venous whole blood. The result of the bile culture submitted before this time was ESBL (Extended-spectrum β lactamase) negative for *Escherichia coli*.

**Fig. 1:**
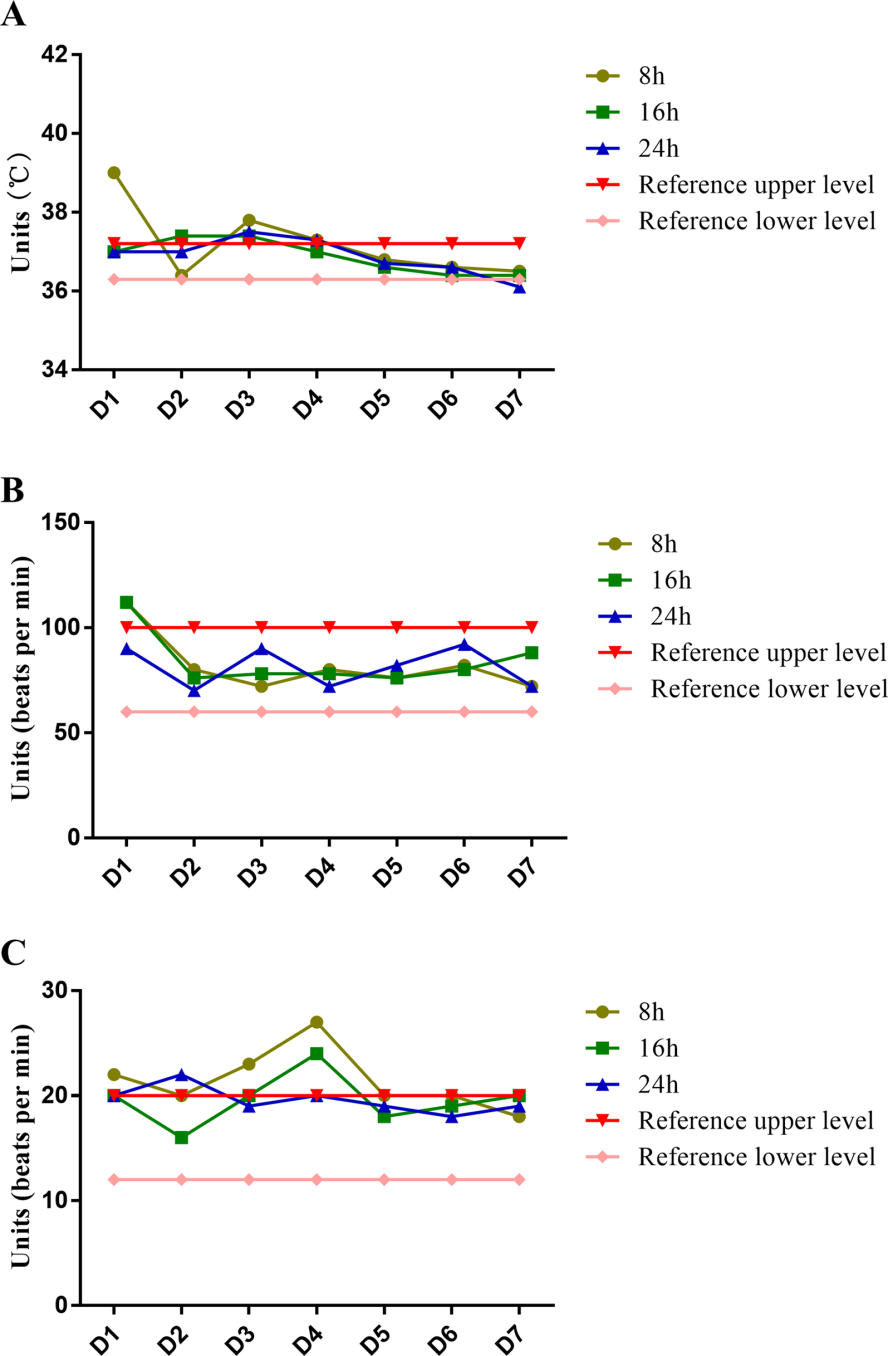
Temperature (**A**), heart rate (**B**), and respiration rate (**C**) changes in a week

The doctor lowered the anti-infective drug gradient based on the susceptibility results and changed imipenem cilastatin to cefoperazone/sulbactam 2 g intravenous drop Q8h. Blood routine results showed an increase in white blood cells to 12.35 (10^9^/L). The patient’s hypersensitivity CRP, procalcitonin, and other infectious indicators have decreased. The patient’s general condition was well and he was transferred back to general surgery. The temperature changes during the week are shown in Fig. 1.

Patient’s blood culture report on the 7th day of admission was identified as *E. tarda* by adsorption/ionization time-of-flight mass spectrometry, which was sensitive to conventional drugs. The patient’s infection symptoms were well controlled, he could eat semi-liquid food, without obvious abdominal distension and discomfort, and the second stool was normal. With the T-tube in place, about 600 mL of golden yellow bile was drawn out every day and he was discharged (Table 2).

**Table 2:** Antibiotic susceptibility of *E. tarda* from blood culture

Antibiotic	MIC (µg/mL)	Antibiotic	MIC (µg/mL)
ABPC	≤ 2 S	IPM/CS	≤ 0.2 S
SBT/ABPC	≤ 2 S	MEM,	≤ 0.2 S
CEZ	≤ 4 S	GM	≤ 1 S
CTT	≤ 4 S	TOB	≤ 1 S
CRO	≤ 1 S	AK	≤ 2 S
CAZ	≤ 0.1 S	CIP	≤ 0.2 S
SCF	≤ 8 S	LEV	≤ 0.1 S
FEP	≤ 0.1 S	MH	≤ 1 S
ATM	≤ 1 S	TGC	≤ 0.5 S
TZP	≤ 4 S	DOX	≤ 0.5 S
TIM	≤ 8 S	SXT	≤ 20 S

*ABPC* Ampicillin, *SBT/ABPC* Sulbactam/Ampicillin, *CEZ* Cefazolin, *CTT* Cefotetan, *CRO* Cefatriaxone, *CAZ* Ceftazidime, *SCF* Cefoperazone/sulbactam, *FEP* Cefepime, *ATM* Aztreonam, *TZP* Piperacillin/Tazobactam, *TIM* Ticarcillin/clavulanic acid, *IPM/CS* Imipenem/Cilastatin sodium, *MEM* Meropenem, *GM *Gentamicin, *TOB* Tobramycin, *AK* Amikacin, *CIP *Ciprofloxacin, *LEV* Levofloxacin, *MH* Minocycline, *TGC* Tigecycline, *DOX* Doxycycline, *SXT* Sulfamethoxazole-Trimethoprim, *S* sensitive

## Discussion and conclusions

*E. tarda* is not a colonizing flora of normal human intestines, and it is reported that it is only detected in 0.0073% of healthy human fecal samples[8]. *E. tarda* is a rare opportunistic pathogen in humans, mainly causing gastroenteritis [2], infectious subdural hematoma[9], bacteremia, epidural abscess wound infection [10], muscle necrosis, tissue abscess[7], meningitis, cholecystitis, endocarditis, osteomyelitis, soft tissue infections, and septicemia [11] in humans. Risk factors for *E. tarda* infection include exposure to the aquatic environment or contact with aquatic animals, such as amphibians or fish, eating habits (raw seafood), hepatobiliary underlying diseases, blood system tumors, etc. At present, there are few cases of *E. tarda* causing bloodstream infections. Once *E. tarda* enters the blood, the patient’s mortality rate is as high as 50% [12]. This paper is a case of septic shock caused by a blood infection of *E. tarda*, which deserves clinical attention. This patient has no history of exposure to the aquatic environment, but he had eaten sashimi before the onset, and then experienced symptoms of pain in the upper right abdomen with chills and fever. Therefore, this paper is considered that the patient’s consumption of infected raw sashimi was one of the factors in the biliary infection. The main drug resistance research of *E. tarda* is mainly colistin, such as polymyxin B and penicillin [13], which are sensitive to most Gram-negative antibiotics. The patient’s susceptibility results suggest sensitivity to most commonly used antibiotics, including carbapenems (ertapenem, imipenem, meropenem), cephalosporins (ceftazidime, ceftriaxone, cefoperazone, cefepime), β-lactamase inhibitors (cefoperazone sodium sulbactam sodium, aminoglycosides (amikacin, tobramycin), quinolones (levofloxacin, ciprofloxacin), tetracyclines (minocycline, doxycycline, tigecycline). The patient, in this case, has common bile duct stones with acute cholecystitis, septic shock, obstructive jaundice hepatitis sanyang, poor basic condition, and *E. tarda*, caused by a series of infections. Fortunately, before the culture results came out, clinicians empirically used imipenem and then bile cultured *Escherichia coli*. The changed cefoperazone sulbactam is also sensitive to *E. tarda*. Therefore, the overall anti-infective treatment effect as well, and the patient’s symptoms of the infection quickly improved. The reported that patients with *E. tarda* generally have underlying diseases, mainly including hepatobiliary diseases (cirrhosis, gallstones and alcohol abuse), malignant tumors (hepatobiliary and gastrointestinal tract) and iron overload status (sickle cells), leukemia, and neonatal status) [12]. In this case, the patient has chronic hepatitis B, gallstones, and bile duct stones, and a poor diet may increase the risk of *E. tarda* infection. In general, clinicians should consider the potential risk factors for *E. tarda*.

To sum up, we report a case of *E. tarda* with acute cholangitis, acute cholangitis, gallstones with acute cholecystitis, and septic shock. Although *E. tarda* is a rare pathogen, it can cause fatal infections similar to those caused by *Aeromonas* and *Vibrio vulnificus* [9]. Avoiding raw or undercooked food is a simple measure to prevent fatal foodborne infections. Clinicians should emphasize the importance of this for patients with potential risk factors.

## Data Availability

Not applicable.

## Abbreviations

*E. tarda*: *Edwardsiella tarda*
ESBL: Extended spectrum β lactamase
CRP: C-reactive protein
